# EGF-coated gold nanoparticles provide an efficient nano-scale delivery system for the molecular radiotherapy of EGFR-positive cancer

**DOI:** 10.3109/09553002.2016.1145360

**Published:** 2016-03-21

**Authors:** Lei Song, Nadia Falzone, Katherine A. Vallis

**Affiliations:** ^a^ CR-UK/MRC Oxford Institute for Radiation Oncology, Department of Oncology, University of Oxford, Oxford, UK; ^b^ Department of Biomedical Science, Tshwane University of Technology, Pretoria, South Africa

**Keywords:** Gold nanoparticles, Auger electron emitters, EGF, Indium-111, targeted radiotherapy

## Abstract

**Purpose** Radiolabeled antibodies and peptides hold promise for molecular radiotherapy but are often limited by a low payload resulting in inadequate delivery of radioactivity to tumour tissue and, therefore, modest therapeutic effect. We developed a facile synthetic method of radiolabeling indium-111 (^111^In) to epidermal growth factor (EGF)-gold nanoparticles (^111^In-EGF-Au NP) with a high payload.

**Materials and methods** EGF-Au NP were prepared *via* an interaction between gold and the disulphide bonds of EGF and radiolabeled using ^111^InCl_3_. Targeting efficiency was investigated by quantitating internalized radioactivity and by confocal imaging following exposure of MDA-MB-468 (1.3 × 10^6^ EGFR/cell) and MCF-7 (10^4^ EGFR/cell) cells to Cy3-EGF-Au NP. Cytotoxicity was evaluated in clonogenic assays.

**Results** The proportion of total administered radioactivity that was internalized by MDA-MB-468 and MCF-7 cells was 15% and 1.3%, respectively (mixing ratio of EGF:Au of 160). This differential uptake in the two cell lines was confirmed using confocal microscopy. ^111^In-EGF-Au NP were significantly more radiotoxic to MDA-MB-468 than MCF-7 cells with a surviving fraction of 17.1 ± 4.4% versus 89.8 ± 1.4% (*p* < 0.001) after exposure for 4 h.

**Conclusions** An ^111^In-labeled EGF-Au nanosystem was developed. It enabled targeted delivery of a high ^111^In payload specifically to EGFR-positive cancer cells leading to radiotoxicity that can be exploited for molecularly targeted radiotherapy.

## Introduction

The term ‘nanomedicine’ refers to the biomedical application of nanostructures and nanomaterials that measure 1–100 nm. Nanomedicine has the potential to significantly impact clinical practice, particularly in the treatment of cancer. Some commentators have predicted that it will change the landscape of pharmaceutics. As well as the enhanced permeability and retention (EPR) effect (Maeda et al. 2000), nanomedicines that incorporate targeting ligands can selectively deliver cytotoxic agents to malignant cells, resulting in therapeutic efficacy with few side effects. Radiolabeled nanosystems have attracted interest for cancer imaging and therapy (Hong et al. 2009; Xing et al. 2014). Molecularly targeted agents, including radiolabeled antibodies and peptides, are commonly limited by a low payload (Hainfeld et al. 2008; Bouchat et al. 2010) and, therefore, insufficient delivery of radioactivity to tumours (Ballot et al. 2006). This may result in therapeutic failure and adverse effects due to the accumulation of radioactivity in and irradiation of normal tissues (Steiner and Neri 2011). Zevalin® (^90^Y-Ibritumomab tiuxetan) showed promising results in the treatment of non-Hodgkin’s lymphoma, partly explained by the inherent sensitivity of lymphoma cells to radiotherapy. However attempts at radioimmunotherapy of solid tumours have been less successful (Steiner and Neri 2011; Eblan and Wang 2013). One strategy to optimize the efficacy of molecularly targeted radionuclide agents is to develop nanoparticle-based targeted delivery systems. By using radiolabeled nanosystems, much higher payloads are achievable due to the large surface area to volume ratio that is typical of nano-based systems. More importantly, a large number of targeting ligands, such as antibodies, peptides or aptamers, can bind to a single nanoparticle, exploiting the multivalent effect. This allows maximal binding to the molecular target in vivo, improving delivery of radioactivity to target tissue with improved imaging quality and therapeutic efficacy. The large surface area of nanoparticles (NP) allows their modification (e.g., via PEGylation) and alteration of their surface properties to improve stability and pharmacokinetics in vivo (Gref et al. 2012). It also offers an opportunity to load a combination of imaging, radiotherapeutic and/or chemotherapeutic moieties for multimodal tumour imaging and therapy (Xing et al. 2014). Radiolabeled or unlabeled antibodies, antibody fragments or peptides have a large volume of distribution in normal tissues, whereas the relatively large size of nanoparticles prevents their penetration through normal vasculature and capillaries, limiting accumulation in normal tissues and minimizing side-effects (Choi et al. 2007; Eblan and Wang 2013).

Au NP are often selected for investigation because of their ease of synthesis and functionalization, monodispersity, controllable size, and non-toxicity. Au NP are promising nanocarriers for the delivery of both small molecule drugs and biomolecules (i.e., nucleic acids, proteins, peptides and carbohydrates) into target tissues (Rana et al. 2012; Song et al. 2013; Cao-Milán and Liz-Marzán 2014). Also, due to their unique physicochemical properties, Au NP have been exploited for phototherapy, as contrast agents and radiosensitizers (Hainfeld et al. 2013; Cao-Milán and Liz-Marzán 2014). Importantly, Au NP-based targeted nanosystems such as AurImmune™ (CYT-6091, Au-rhTNF) (Libutti et al. 2010) and AuroShell^®^ (gold-silica nanoshells) (Ventola 2012), have progressed to clinical trials.

**Figure 1.**
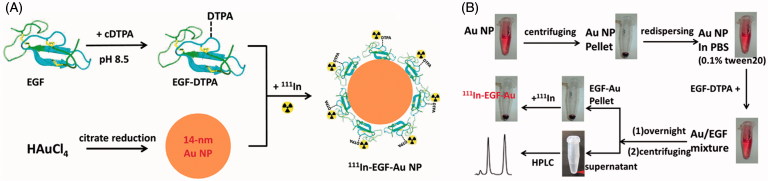
(A) Schematic illustration of the preparation of ^111^In-EGF-Au NP. Au NP were synthesized via the citrate reduction method and then conjugated with chelating ligand DTPA-coupled EGF to form EGF-Au NP. After purification, the EGF-Au NP were radiolabeled with ^111^In to produce ^111^In-EGF-Au NP. (B) Detailed procedures to synthesize ^111^In-EGF-Au NP.

^111^In, a radionuclide that is commonly used for single-photon emission computed tomography (SPECT) imaging, emits Auger electrons that have the potential to kill cancer cells when localized in nuclei or close to sensitive extra-nuclear targets (i.e., cell membrane and mitochondria) (Freudenberg et al. 2014; Pouget et al. 2015). This is primarily due to the short effective radiation range of the Auger electrons. Here, we report a facile but effective method for synthesis of ^111^In-labeled Au NP with a large radioisotope payload for molecularly targeted radiotherapy (Figure 1). Epidermal growth factor (EGF) was selected as the targeting ligand, and was coupled with diethylenetriaminepentaacetic dianhydride (cyclic DTPA anhydride) to form a chelating ligand modified EGF (DTPA-EGF) for subsequent labeling with ^111^In. The DTPA-EGF conjugate has been intensively studied by our group and others as an effective method for ^111^In delivery for radiotherapy (Reilly et al. 2000; Chen et al. 2003; Hua et al. 2007; Cornelissen et al. 2011; Vallis et al. 2014). This approach depends on nuclear translocation of EGFR after binding of radiolabeled-EGF leading to nuclear localization of radioactivity (Lin et al. 2001; Hua et al. 2007).

Au NP have been exploited for the attachment of biomolecules including oligonucleotides (Rosi et al. 2006; Song et al. 2013), peptides (Paciotti et al. 2004; Joshi et al. 2006) and antibodies (Ackerson et al. 2006). These biomolecules can bind to Au NP through accessible thiol, disulphide and amine groups. Although EGF has three disulphide bonds, the effect of the tertiary structure of EGF on accessibility of these groups to Au was unknown. We explored the possibility of using the disulphide bonds of EGF for EGF-Au preparation via Au-S bonds. This offers a simple method for preparation of an ^111^In-EGF nanosystem with a high payload of radioactivity per Au NP; facilitates future possible surface modifications (e.g., PEGylation via thiol-PEG); and opens up the possibility of exploiting Au NP as radiosensitizers by combining them with external-beam radiation. For therapeutic applications, it is desirable to attach as many EGF as possible to each Au NP to maximize ^111^In loading and, therefore, radiotoxicity. However, it is also important to take into account the possible steric effects of dense EGF loading that could cause reduced targeting efficiency and radiotoxicity. The purpose of the current study was to develop a facile method for attachment of radiolabeled EGF to 14 nm Au-NP for the specific targeting of EGFR-expressing breast cancer cells.

## Materials and methods

### Synthesis of Au NP

Au NP (14 ± 2 nm) were synthesized by citrate reduction of Hydrogen tetrachloroaurate(III) hydrate (HAuCl_4_; Alfa Aesar, Heysham, Lancashire, UK). Briefly, 80 mg HAuCl_4_ was dissolved in 200 ml of water and heated to reflux. Then 228 mg trisodium citrate (Sigma-Aldrich, Dorset, UK) dissolved in 20 ml of water was added and the resulting solution was continuously refluxed for 20 min. The resulting Au NP were characterized by a UV-vis spectrophotometer (6505, Jenway Ltd, Essex, UK) and the concentration was calculated using the Beer-Lambert law (ɛ = 2.4 * 10^8^ M^−1^·cm^−1^).

### Synthesis of DTPA-EGF

Synthesis of DTPA-EGF was adapted from a published method (Reilly and Gariepy 1998). Briefly, 1 mg EGF (Peprotech, London, UK) was dissolved in 1 ml of 0.1 M sodium bicarbonate buffer (pH 8.5) and reacted with a 5-fold molar excess of cyclic DTPA anhydride (Sigma) (dissolved in dry DMSO) at room temperature for 1 h to produce DTPA-coupled EGF. DTPA-EGF was purified by size exclusion chromatography (Sephadex G25 mini-column, pH 7.4 PBS as elution buffer).

### Preparation of EGF-Au NP

Au NP (1 ml) were centrifuged (13,000 rpm, 30 min) to obtain Au NP pellets. Pellets were redispersed in PBS (0.2 ml containing 0.1% tween 20) and then mixed with 4, 7.5, 15, 30, 45, 60, 75 or 105 *μ*l of EGF-DTPA (40 nmol/ml) respectively (the mixing molar ratios of DTPA-EGF to Au NP are 10, 20, 40, 80, 120, 160, 200 and 280). The resulting mixtures were incubated at 4 °C overnight. The prepared DTPA-EGF-Au NP were centrifuged to remove free unbound EGF-DTPA and redispersed in 0.1 M sodium citrate (pH 5.5). The amount of EGF attached to Au NP was calculated by determining free unbound EGF-DTPA via reversed-phase HPLC (Waters 2695, Milford, MA, USA; C18 column, 5–90% acetonitrile [0.1% TFA] 30 min, 100 *μ*l injection, detecting wavelength 220 nm, room temperature). The retention time of EGF-DTPA was 9.5 min. A standard curve of EGF-DTPA was generated and used to determine the concentration of unbound EGF-DTPA.

### ^111^In-radiolabeling of EGF-Au NP

EGF-Au NP (dispersed in 0.1 M sodium citrate, pH 5.5) were incubated with ^111^InCl_3_ (Perkin Elmer, Boston, MA, USA) for 1 h at room temperature (specific activity: 37.5 MBq/nmol EGF [i.e., 6 MBq/*μ*g EGF]), resulting in ^111^In-EGF-Au NP. Quality control was performed by ITLC, Phosphor imaging (Cyclone Plus storage phosphor system, Perkin Elmer) and size exclusion chromatography. The synthesis of ^111^In-EGF-Au NP is summarized in Figure 1.

### Synthesis of Cy3-EGF and Cy3-EGF-Au NP

A vial of Cy3-NHS ester (GE Healthcare, Little Chalfont, Buckinghamshire, UK) was dissolved in 8 *μ*l of dry DMSO and mixed with 500 *μ*l of EGF (150 nmol/ml in 0.1 M sodium bicarbonate buffer, pH 8.5) in darkness for 2 h (room temperature) to synthesize Cy3-labeled EGF. Cy3-EGF was then separated from excess, unconjugated Cy3 by SEC (Sephadex G25 mini-column, pH 7.4 PBS as elution buffer), the concentration of which was calculated using the Beer-Lambert law (ɛ_Cy3_ = 1.5 * 10^5^ M^−1^·cm^−1^). Cy3-EGF-Au NP were prepared using the same method described above using a molar mixing ratio of Cy3-EGF:Au of 160.

### *Stability of ^111^In-EGF-Au NP in PBS and fetal bovine serum* (*FBS*)

^111^In-EGF-Au NP were incubated with PBS or FBS (Invitrogen, Paisley, UK) for 8 h at 37 °C and then analyzed by size exclusion HPLC (Waters 2695) with both UV and radio detectors for testing transchelation of ^111^In to serum proteins (HPLC mobile phase: pH 7.4 PBS; flow rate: 0.8 ml/min; detection wavelength: 280 nm).

### Internalization assay

MDA-MB-468 and MCF-7 cells were seeded in 24-well plates overnight (2 × 10^5^ cells/well). ^111^In-EGF-Au NP ([EGF] = 40 nM in 200 *μ*l DMEM, specific activity: 37.5 MBq/nmol EGF) or equivalent amounts of ^111^InCl_3_ (i.e., 0.3 MBq in 200 *μ*l DMEM) were added to each well. After incubation for 4 h, the medium containing ^111^In-EGF-Au NP or ^111^InCl_3_ was removed and cells were washed using 0.1 M glycine·HCl (pH 2.5) to remove cell surface bound radioactivity. Cells were then lyzed using 0.1 M NaOH and the internalized radioactivity was counted using an automated gamma counter (Wizard, Perkin Elmer).

### Competitive binding assay

MDA-MB-468 and MCF-7 cells were seeded in 24-well plates overnight (2 × 10^5^ cells/well) and exposed to ^111^In-EGF-Au NP (mixing ratio of EGF:Au = 160; [EGF] = 10 nM, specific activity: 37.5 MBq/nmol) with increasing amounts of cold, unlabeled EGF. After 4 h incubation at 37 °C, the cells were washed with PBS and lyzed using 0.1 M NaOH and radioactivity counted.

### Confocal microscopy

MDA-MB-468 and MCF-7 cells were seeded in a Lab-Tek chamber slide overnight and exposed to Cy3-EGF-Au NP ([EGF] = 40 nM). After 4 h or 12 h incubation at either 4 °C or 37 °C, cells were washed twice with PBS, fixed using 4% formaldehyde for 10 min at room temperature and then mounted using Vectashield mounting medium with DAPI. The cells were imaged on a Zeiss 530 microscope (Zeiss, Welwyn Garden City, UK).

### Clonogenic assay

MDA-MB-468 and MCF-7 cells were seeded in 96-well plates overnight (5 × 10^3^ cells/well) and treated with ^111^In-EGF-Au NP ([EGF] = 40 nM in 200 *μ*l DMEM, specific activity: 37.5 MBq/nmol) or equivalent amounts of ^111^InCl_3_. After 4 h incubation, cells were trypsinized and seeded in T25 flasks with fresh DMEM (5 ml). After 10- to 14-day incubation, the flasks were washed using PBS and cell colonies were stained using methylene blue (2% methylene blue in methanol/water [1:1]) and counted.

### Optical microscopy

MDA-MB-468 and MCF-7 cells were seeded in 96-well plates overnight (5 × 10^3^ cells/well) and incubated with ^111^In-EGF-Au NP (mixing ratio of EGF/Au = 160, [EGF] = 40 nM, specific activity: 37.5 MBq/nmol) for 4 h followed by incubation in fresh medium for 44 or for 48 h. Cells were imaged on a Nikon Eclipse E800 microscope (Surrey, UK).

## Results

### Synthesis of EGF-Au NP

**Figure 2.**
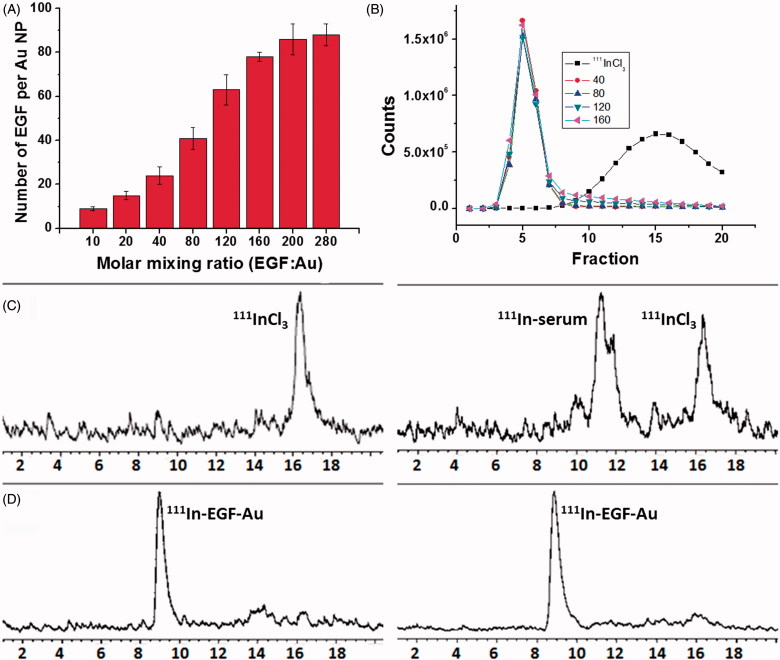
(A) EGF loading as a function of the molar mixing ratio of DTPA-EGF to Au NP. Results are expressed as the mean ± SD of three independent experiments. (B) G50 size-exclusion chromatography (SEC) of ^111^InCl_3_ and ^111^In-labeled EGF-Au NP generated using mixing ratios of 40, 80, 120 and 160, respectively, showing that EGF-Au NP for each mixing ratio were successfully radiolabeled with ^111^In. (C) Stability of ^111^InCl_3_ in PBS (left panel) and FBS (right panel) showing that a large portion of ^111^In was associated with proteins in FBS. (D) Stability of ^111^In-EGF-Au (EGF:Au = 160) in PBS (left panel) and FBS (right panel) performed by size exclusion HPLC with a radio detector, showing no ^111^In-transchelation to serum proteins.

DTPA-EGF was attached to Au NP via the Au-S bond. The number of DTPA-EGF attached to each Au NP was calculated by determining free unbound EGF via HPLC (Figure 2A). For example, the attached number of EGF increased from 24 ± 5 to 78 ± 3 when the mixing ratio of EGF:Au was raised from 40 to 160. However the amount of EGF loading was only increased slightly when the mixing ratio was increased from 160 to 280. The highest EGF density that can be achieved using this method is approximately 78 EGF per Au NP. To select the optimal EGF loading, EGF-Au NP with mixing ratios (EGF:Au) of 40, 80, 120 and 160 were radiolabeled and then tested *in vitro*.

### ^111^In-radiolabeling of EGF-Au NP

^111^In-radiolabeling of EGF-Au was confirmed by size-exclusion chromatography (SEC) using a Sephadex G50 mini-column (Figure 2B) and instant thin layer chromatography (ITLC) (Supplementary Figure S1, available online). ^111^In-EGF-Au NP were successfully prepared with a radiolabeling yield (RLY) higher than 90% for all mixing ratios. Neither ^111^In-EGF nor ^111^In was found in the ^111^In-EGF-Au samples (based on size exclusion HPLC with UV and radio detectors). This indicates that both DTPA-EGF and ^111^In were attached to Au NP. The stability of ^111^In-EGF-Au NP was tested in FBS for 8 h at 37 °C. No ^111^In-transchelation to serum proteins was observed. In contrast, more than 70% of free ^111^In became associated with serum proteins (Figure 2C and D; Supplementary Figure S2 [available online] using a mixing ratio of 160 as an example).

### Cellular studies

**Figure 3.**
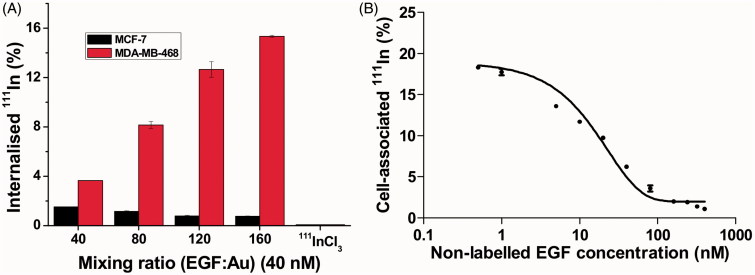
(A) Cellular internalization of ^111^In-EGF-Au NP of varying EGF/Au ratio (all containing 40 nM EGF) and ^111^InCl_3_ at 4 h. Results are expressed as the mean ± SD of three independent experiments. (B) Competitive binding assay: Increasing concentrations of unlabeled EGF were incubated with MDA-MB-468 cells and ^111^In-EGF-Au NP (mixing ratio: 160; containing EGF concentration of 10 nM).

To investigate whether EGF retains affinity for EGFR when it is incorporated into ^111^In-EGF-Au NP, internalization assays were performed using a gamma counter to measure radioactivity in MDA-MB-468 and MCF-7 cells. Cells were exposed to ^111^In-EGF-Au NP (specific activity: 37.5 MBq/nmol) for 4 h. The uptake of ^111^In was greater in MDA-MB-468 compared to MCF-7 cells (Figure 3A). Furthermore, the higher the mixing ratio used for NP preparation, the greater the uptake of radioactivity by MDA-MB-468. For example, when cells were exposed to ^111^In-EGF-Au NP generated using a mixing ratio of 160, more than 15% of total administered radioactivity was internalized by MDA-MB-468 cells while only 0.75% accumulated in MCF-7 cells. There was very little uptake of ^111^InCl_3_ by either cell line (i.e., 0.06% and 0.07% for MDA-MB-468 and MCF-7, respectively).

To validate that the high uptake by MDA-MB-468 cells was the result of EGF-EGFR interaction, a competitive binding assay was performed (Figure 3B). MDA-MB-468 cells incubated with non-radiolabeled EGF (0–400 nM) were mixed with ^111^In-EGF-Au NP (10 nM; mixing ratio of 160). As expected, the uptake of ^111^In-EGF-Au NP was inhibited by increasing concentrations of non-labeled EGF.

**Figure 4.**
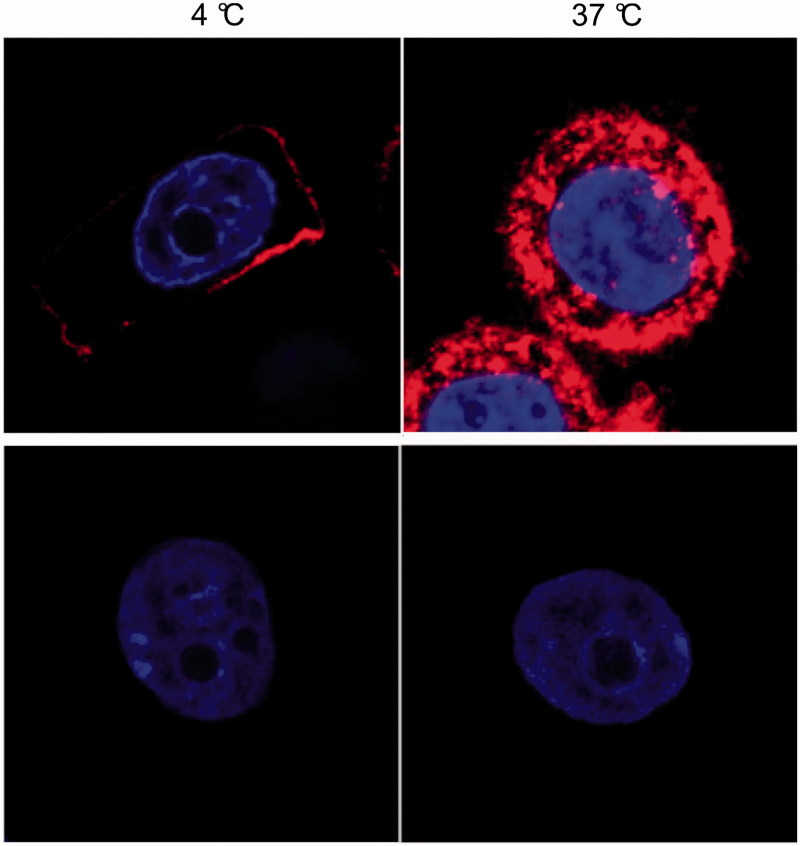
Representative confocal microscopy images of MDA-MB-468 (upper images) and MCF-7 (lower images) cells after incubation for 4 h with Cy3-EGF-Au NP ([EGF] = 40 nM) at 4 °C and 37 °C.

Confocal microscopy was used to visualize the interaction of EGF with EGFR at 4 °C and 37 °C using Cy3-modified EGF-Au NP (Cy3-EGF-Au; mixing ratio of EGF:Au, 160) (Figure 4). After incubation for 4 h, Cy3 fluorescence was observed exclusively on the membrane of the MDA-MB-468 cells at 4 °C and inside the cells at 37 °C but was not detectable in MCF-7 cells at either temperature. Furthermore, Z-stack profiles of MDA-MB-468 cells (Supplementary Figure S3) showed focal intranuclear localization of Cy3.

**Figure 5.**
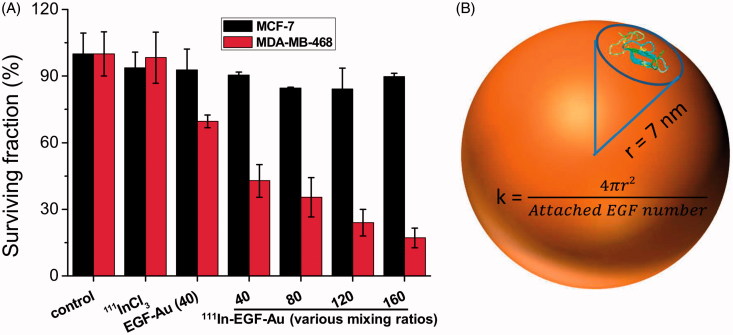
(A) Clonogenic survival of MDA-MB-468 and MCF-7 cells after 4 h exposure to ^111^In-EGF-Au (Mixing ratios, 40–160; [EGF] = 40 nM; specific activity: 37.5 MBq/nmol) or equivalent amounts of ^111^InCl_3_ or unlabeled EGF-Au with mixing ratio of 40. Results are expressed as the mean ± SD of three independent experiments. (B) Model of an EGF-Au NP used to calculate the footprint (k) of EGF that varies depending on the number of EGF attached per Au NP.

The radiotoxicity of ^111^In-EGF-Au NP was tested in clonogenic assays. It is evident from Figure 5(A) that both non-radiolabeled EGF-Au and ^111^In-EGF-Au NP were non-toxic to MCF-7 cells. The control treatment, ^111^InCl_3_, was not toxic to either cell line. Non-labeled EGF-Au (mixing ratio of 40) reduced the surviving fraction (SF) to 69.6 ± 2.8%. However the addition of ^111^In to give ^111^In-EGF-Au NP (mixing ratio of 40) was more toxic (SF 42.8 ± 7.3%). Furthermore, greater radiotoxicity was observed after increasing the mixing ratio (EGF loading). For example, when treated with ^111^In-EGF-Au NP at mixing ratio of 160, the SF of MDA-MB-468 cells was 17.1 ± 4.4%, 2.5-fold lower than mixing ratio of 40. The selective toxicity of ^111^In-EGF-Au NP is shown in Supplementary Figure S5.

### Microdosimetry

To correlate SF results with ^111^In-EGF-Au NP internalization, a microdosimetric approach based on the Medical Internal Radiation Dose (MIRD) formulism (Goddu and Budinger 1997) was taken to evaluate single cell dose. According to the MIRD formulism, the radiation dose (*D_r_*) in a target region *r_k_*, is the product of the cumulated activity Ã_h_ resulting from a source region *r_h_*, and the mean absorbed dose to the target region per unit cumulated activity in the source region (*S*), i.e.,
(1) 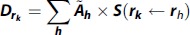



*S*-values were derived from event-by-event Monte Carlo (MC) simulations with the general purpose MC code PENELOPE 2011 (PENetration and Energy Loss of Positrons and Electrons) (Salvat et al. 2011), with the radiation spectra of ^111^In taken from the unabridged nuclear decay data (RADTABS software Ver. 2.2) (Eckerman and Endo 2008) (Supplementary Table S1, available online). As many of the low energy Auger electrons emitted by ^111^In have ranges equivalent to the dimensions of the Au NP, self-absorption, i.e., the fraction of emitted electron energy (per decay) absorbed within the Au NP, was determined by MC simulation. For dose calculations in MDA-MB-468 cells, three scenarios were considered: (1) Activity (^111^In-EGF-Au NP at a mixing ratio of 160) is homogenously distributed on the cell surface (Cs); (2) throughout the cytoplasm (Cy); and (3) perinuclear (PN), to closely simulate experimental results of ^111^In-EGF-Au NP distribution after 4 and 24 h incubations (Supplementary Table S1).

Self-absorption resulted in a 2% decrease in activity emitted by the radiolabeled Au NP, therefore assuming that all activity (37.5 MBq/nmol; 40 nM) after the 4 h incubation was uniformly distributed throughout the cytoplasm (only taking physical decay into consideration), this will result in a nuclear dose of 0.25 Gy/cell. This dose will increase almost 9-fold, with a perinuclear distribution of activity after 24 h. Nuclear doses, comparing ^111^In-EGF-Au NP accumulation after 4 h in MDA-MB-468 (0.23 Bq/cell) with MCF-7 (0.01 Bq/cell) cells, show an almost 6-fold difference in dose.

### Modeling of EGF loading on Au NP

To investigate the possibility that higher EGF loading might cause steric effects on EGF-EGFR binding, and also to further interpret the observation that targeted ^111^In-EGF-Au NP with the highest EGF loading shows the greatest internalization and radiotoxicity, a concept of footprint (k) (Hill et al. 2009) was introduced for modeling (Figure 5B). It is defined here as the average area occupied (or shared) by one EGF molecule on the Au NP surface. This can be used to understand the spatial arrangement of EGF on the Au surface. Assuming that the EGF is spherical in shape and evenly distributed on the surface of a perfectly spherical Au NP, the footprint of one EGF in the ^111^In-EGF-Au NP (mixing ratio of 160) can be calculated by dividing the Au surface area (4πr^2^, r is the radius of Au NP [7 nm]) by the number of attached EGF per Au (78), which is 7.9 nm^2^. Based on the estimated volume of EGF itself (approximately 7500 A^3^), the radius of EGF (r_EGF_) is 1.2 nm and its orthographic projection (πr^2^) is calculated as 4.5 nm^2^, 1.75-fold difference from its footprint.

## Discussion

A direct binding method was used to synthesize EGF-Au conjugate. The results show that the disulphide groups of EGF can be exploited for binding to Au NP (Figure 2A). We found that the mixing molar ratio of EGF to Au NP was a major determinant of the EGF loading number per NP. The higher the mixing ratio (ranging from 10–160), the greater the EGF loading achieved. Despite further increasing the mixing ratio (>160), the binding of EGF to Au NP saturated at mixing ratio of ∼160. This was also seen or exploited by others to synthesize Au-oligo conjugates with various oligo densities (Li et al. 2013) or to synthesize Au-TNF conjugate, where TNF binding reached saturation at a specific mixing ratio (Paciotti et al. 2004).

^111^In-EGF-Au NP with four different mixing ratios were used for further *in vitro* studies. Internalization assays showed a statistically significant difference (*p* < 0.001) in uptake between MDA-MB-468 and MCF-7 cells. This reflects the difference in EGFR expression (10^2^-fold difference [Reilly et al. 2000]) of MDA-MB-468 versus MCF-7 cells. It also indicates that when EGF is attached to Au NP it retains affinity for EGFR and agrees well with the notion that uptake of ^111^In-EGF-Au NP by MDA-MB-468 is determined by EGF-EGFR binding leading to internalization. When NP are prepared with a high mixing ratio (e.g., 160), EGF and ^111^In loading per Au NP are high, resulting in enhanced uptake. In contrast, there was a much lower uptake of ^111^In-EGF-Au NP by MCF-7 cells with the highest uptake observed when the lowest mixing ratio was used. This suggests that uptake in the EGFR negative cell line is through non-specific internalization. The competitive binding assay further confirmed that EGF retains binding affinity for EGFR when incorporated in Au NP. The 160 mixing ratio was selected for this assay for two reasons: It conferred the highest uptake and resulted in saturation of EGF binding to Au NP, avoiding interaction of non-labeled EGF with ^111^In-EGF-Au NP.

The confocal results are consistent with the observed higher uptake of radioactivity by MDA-MB-468 than MCF-7 cells when exposed to ^111^In-EGF-Au NP. The Z-stack profiles indicate that some Cy3-EGF-Au NP were transported into the MDA-MB-468 nucleus. This agrees well with other reports showing that NP can be carried to nuclei via EGFR nuclear translocation (Yokoyama et al. 2011; Yuan et al. 2013). It is also noted that a considerable portion of internalized Cy3-EGF-Au was located in the perinuclear region after incubation overnight (Supplementary Figure S4). This distribution would enhance the radiotoxicity of ^111^In-EGF-Au NP, as electrons emitted in the perinuclear area contribute to nuclear and, therefore, DNA radiation dose (Hoang et al. 2012).

Clonogenic assays show that non-labeled EGF-Au (mixing ratio of 40) is toxic to MDA-MB-468. High concentration EGF has been reported previously to be toxic to MDA-MB-468 cells (Reilly et al. 2000). However, the therapeutic efficacy was greatly enhanced through radiolabeling. Figures 5(A) shows that ^111^In-EGF-Au NP are selectively radiotoxic to EGFR-positive MDA-MB-468 cells. Microdosimetry showed that the observed difference in SF between MDA-MB-468 and MCF-7 cell lines (Figure 5A – mixing ratio of 160) correlates with the internalization result.

By comparing the footprint and orthographic projection of EGF, it can be seen that a gap exists between neighbouring EGF molecules when attached to the surface of Au NP. As ^111^In-EGF-Au NP generated using a mixing ratio of 160 showed greater internalization and radiotoxicity compared to ^111^In-EGF-Au NP at lower mixing ratios, we assume that the gap between adjacent EGF molecules on Au NP is large enough to avoid steric effects on EGF-EGFR binding.

In summary, a new ^111^In-labeled EGF-Au nanosystem was developed using a facile preparation process. The direct attachment of EGF to Au NP does not perturb EGF-EGFR binding. ^111^In-labeled EGF-Au NP hold promise as a new approach to the treatment of EGFR-positive cancers.

## Supplementary Material

Supporting_information - figures and tables
