# Supplementary material for: EGF-coated gold nanoparticles provide an efficient nano-scale delivery system for the molecular radiotherapy of EGFR-positive cancer
Source: Int J Radiat Biol. 2016 Mar 21;92(11):716–23. doi: 10.3109/09553002.2016.1145360 (PMC5116916; doi:10.3109/09553002.2016.1145360)
Supplement: Supporting_information - figures and tables [file irab_a_1145360_sm3694.doc]

**Supporting information**

**
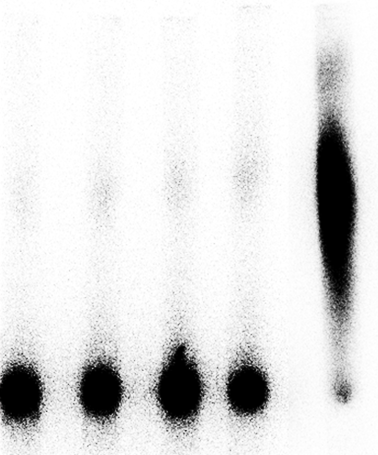
**

**Figure S1.** Phosphor images of ^111^InCl_3_ and ^111^In-EGF-Au NPs (Samples from right to left: ^111^InCl_3_ and ^111^In-EGF-Au NPs generated using the molar mixing ratio of 40, 80, 120 and 160, respectively; ITLC running direction: from bottom to top).


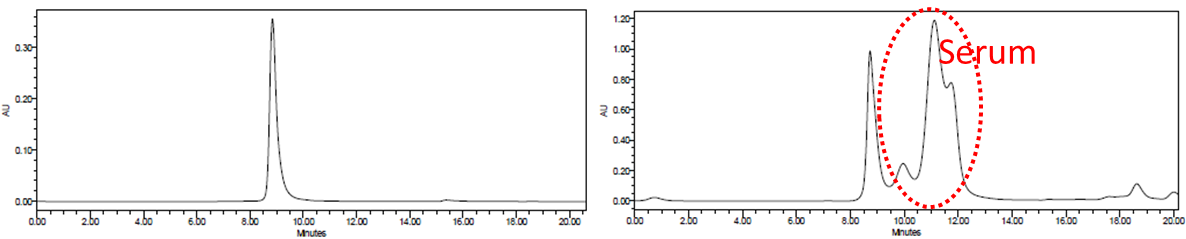


**Figure S2.** HPLC profiles of ^111^In-EGF-Au (EGF:Au=160) in PBS (left panel) and FBS (right panel) performed by size exclusion HPLC with a UV detector.

**
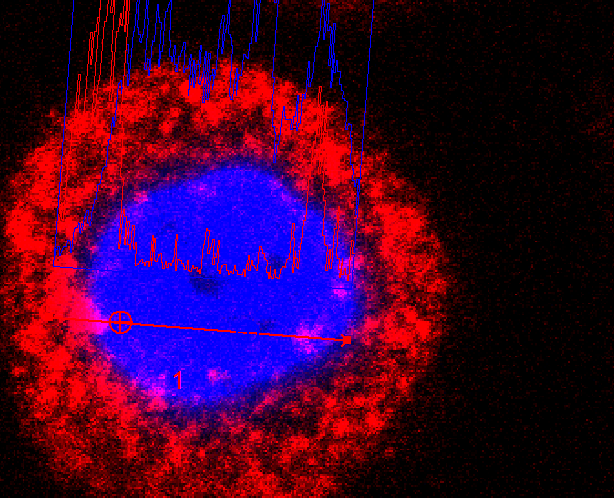

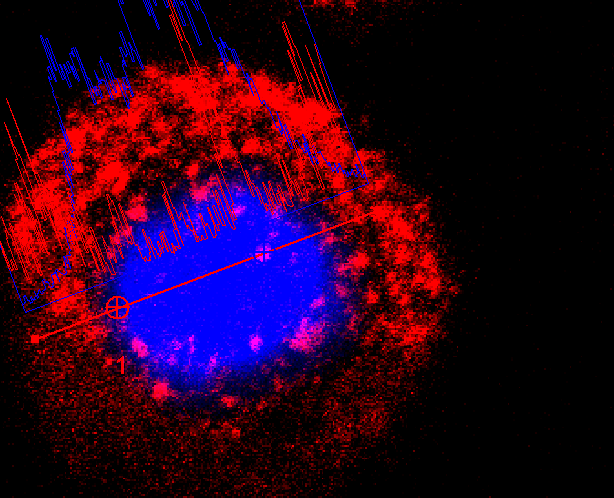
**

**Figure S3.** Two Z-stack profiles of MDA-MB-468 cells after 4 h incubation with Cy3-EGF-Au NPs ([EGF] = 40 nM) at 37 °C showing focal intranuclear localization of Cy3.


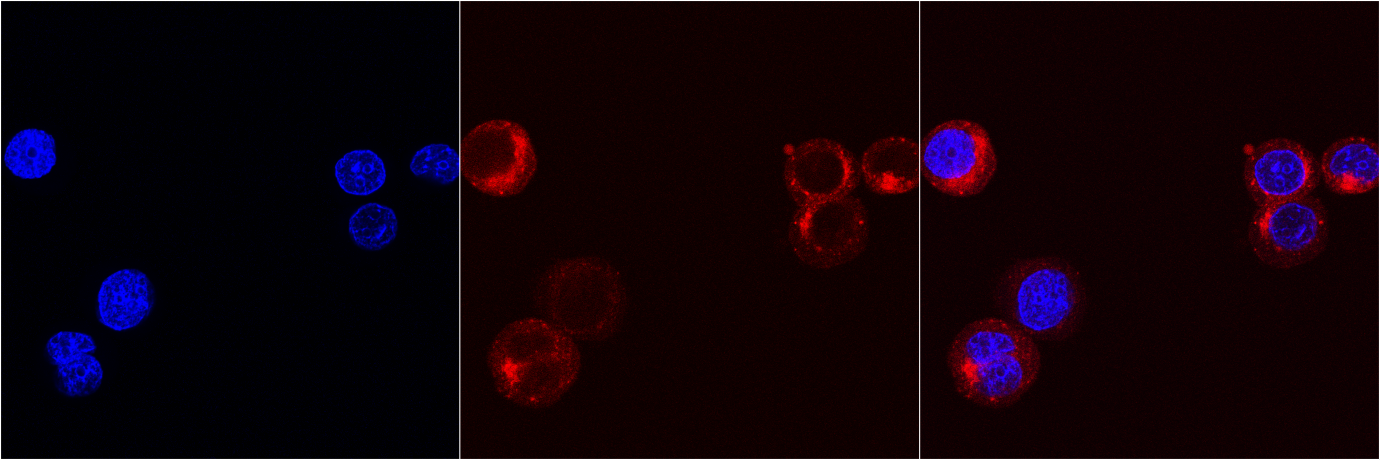


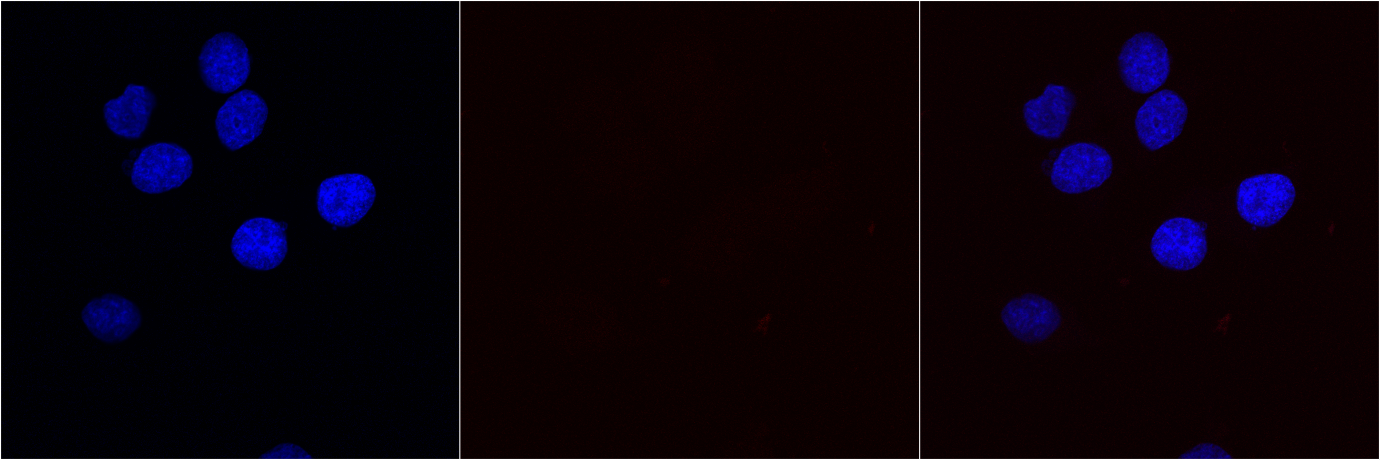


**Figure S4.** Confocal microscopy images of MDA-MB-468 (upper panel) and MCF-7 (lower panel) treated with Cy3-EGF-Au NPs ([EGF] = 40 nM) overnight at 37 °C.


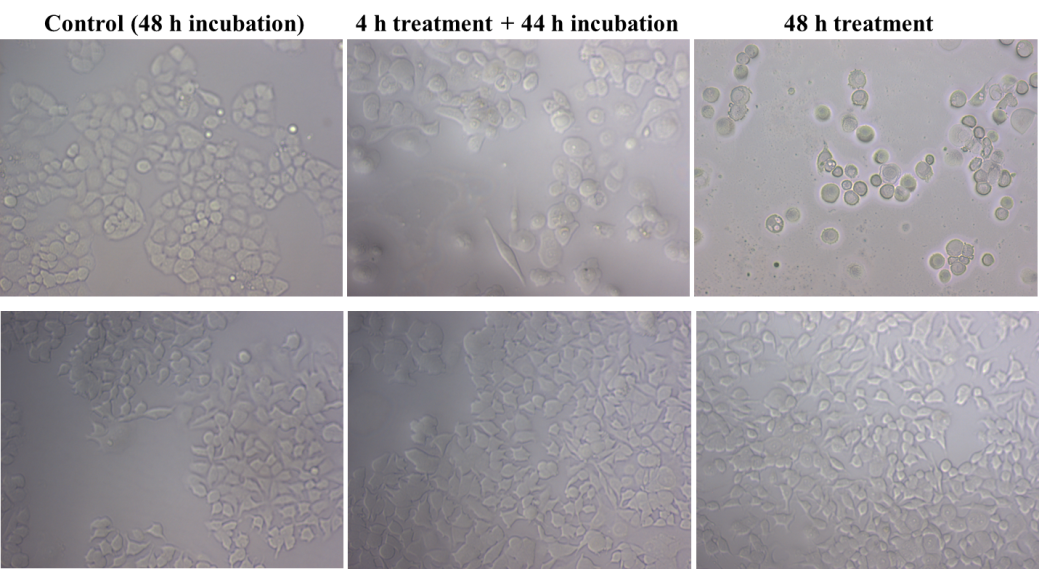


**Figure S5.** Microscope images of MDA-MB-468 (upper panel) and MCF-7 (lower panel) cells treated with 40 nM ^111^In-EGF-Au NPs (mixing ratio, 160), showing that ^111^In-EGF-Au NPs are selectively radiotoxic to MDA-MB-468 cells. From left to right: control without treatment (48 h incubation); 4 h treatment plus 44 h further incubation; 48 h treatment.

**Table SI.** Single cell dosimetry: Summary of *S*-values and comparison of activity distribution (Cs, Cy or PN) and dose (D) to the nucleus.

| **Cell line** | ***S*(N←C)** | ***S*(N←Cs)** | ***S*(N←Cy)** | ***S*(N←PN)** | **MDA-MB-468** | **Dose Ratio** |
| --- | --- | --- | --- | --- | --- | --- |
| MDA-MB-468^1^ | 2.83E-4 | 3.32E-5 | 8.11E-5 | 1.43E-4 | D(Cy)/D(Cs)^a^ | 2.4 |
| MCF-7^2^ | 3.18E-4 | 5.44E-5 | 8.02E-5 | - | D(PN)/D(Cy)^b^ | 8.9 |

*C = cell, N = Nucleus

^1^Rc = 9.8 ± 0.9; Rn = 6.0 ± 0.4

^2^Rc = 9.2 ± 1.4; Rn = 7.6 ± 1.1

^a^4 h incubation; ^b^24 h incubation
